# Supplementary material for: A comprehensive scoping review of intergenerational dance programmes for cohorts with a generational gap
Source: PLoS One. 2024 Dec 19;19(12):e0311564. doi: 10.1371/journal.pone.0311564 (PMC11658520; doi:10.1371/journal.pone.0311564)
Supplement: S2 File — (DOCX) [file pone.0311564.s002.docx]

S1 [dance[Title/Abstract] OR dancer[Title/Abstract] OR dancing[Title/Abstract] OR (movement to music[Title/Abstract])]

S2 [intergenerational[Title/Abstract] OR (older adults and children[Title/Abstract]) OR ((grandparent* OR grandfather OR grandmother) and grandchild*[Title/Abstract])]

S3 [S1 AND S2]
