# Supplementary material for: A comprehensive scoping review of intergenerational dance programmes for cohorts with a generational gap
Source: PLoS One. 2024 Dec 19;19(12):e0311564. doi: 10.1371/journal.pone.0311564 (PMC11658520; doi:10.1371/journal.pone.0311564)
Supplement: S3 File — (DOCX) [file pone.0311564.s003.docx]

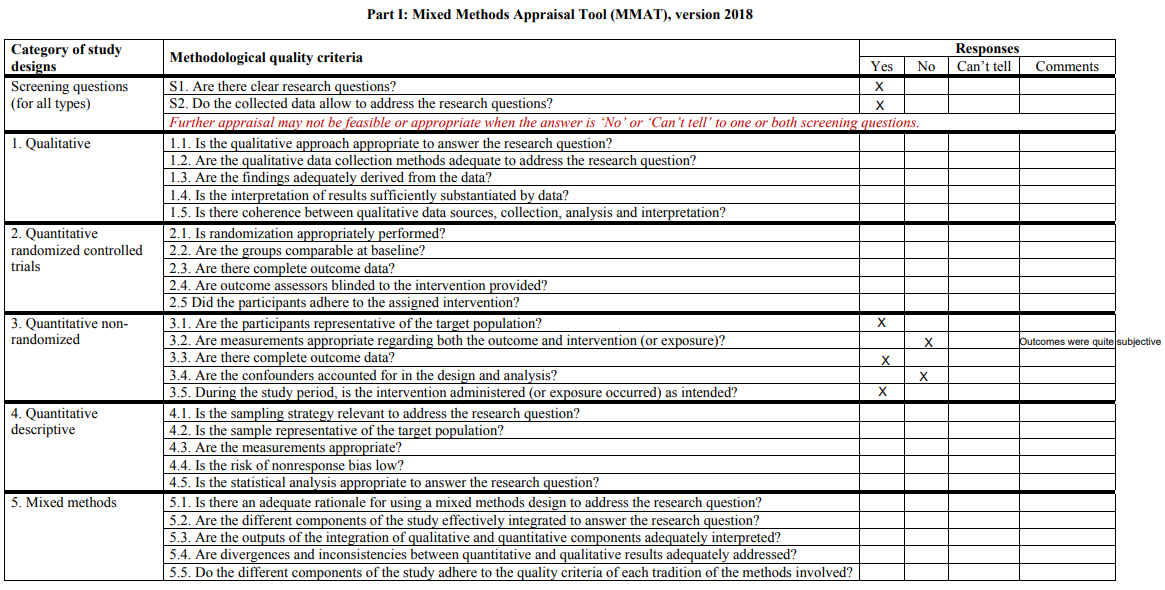


Interactive programs with preschool children bring smiles and conversation to older adults: time-sampling study (Morita and Kobayashi, 2013)


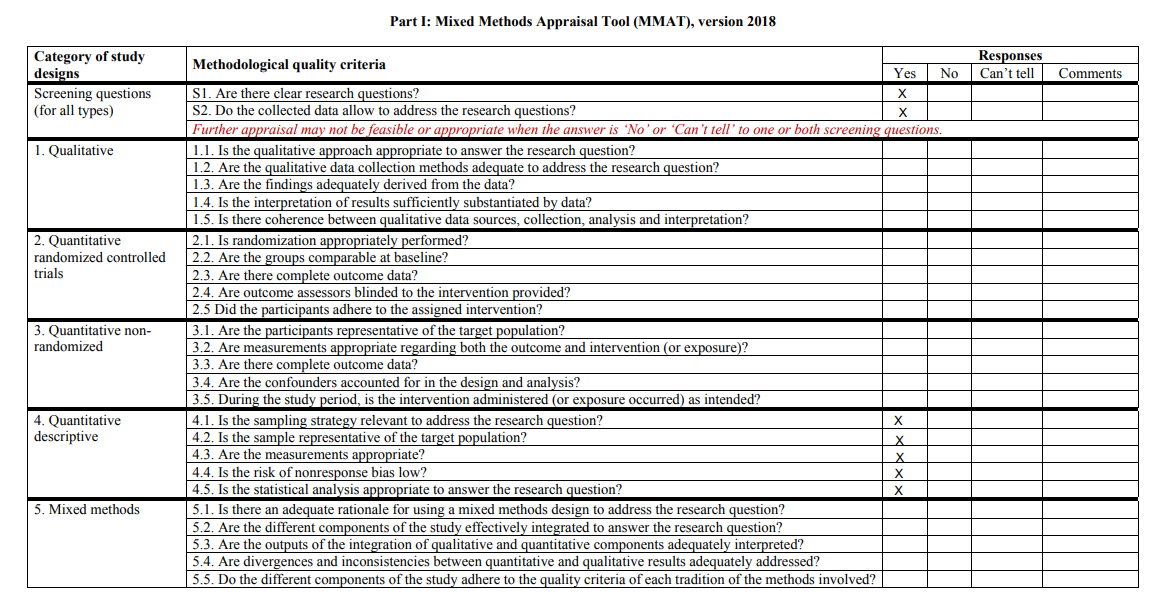


A Survey of Intergenerational Programs in Australian Residential Aged Care Homes During the COVID-19 Pandemic (D’Cunha *et al*. 2023)


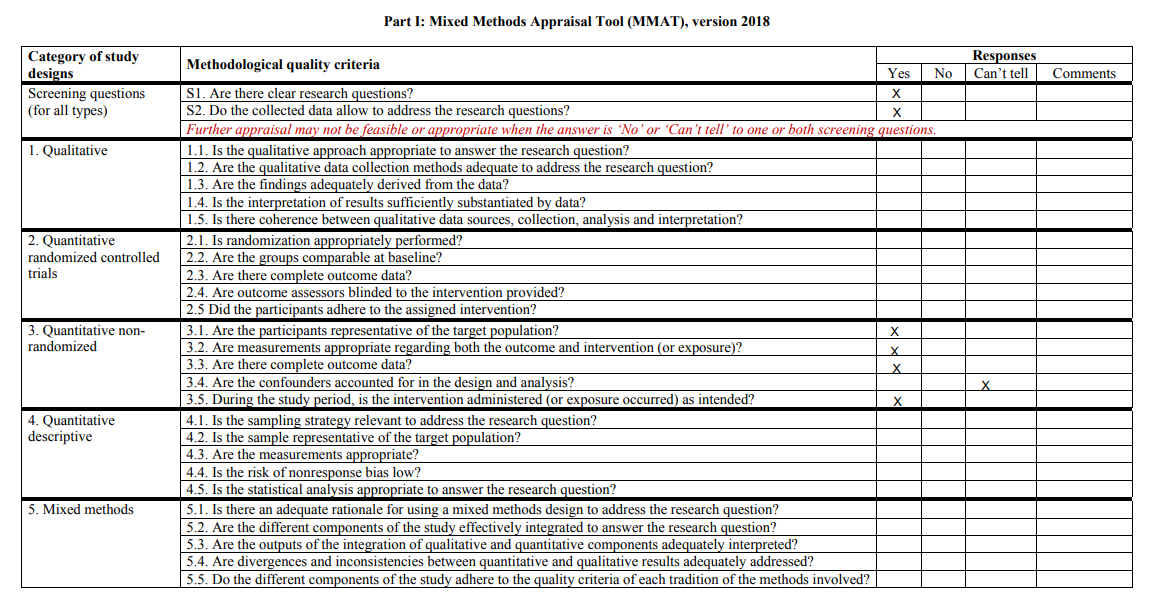


An intergenerational creative dance program for children and frail older adults (Rossberg Gempton and Poole, 2000)


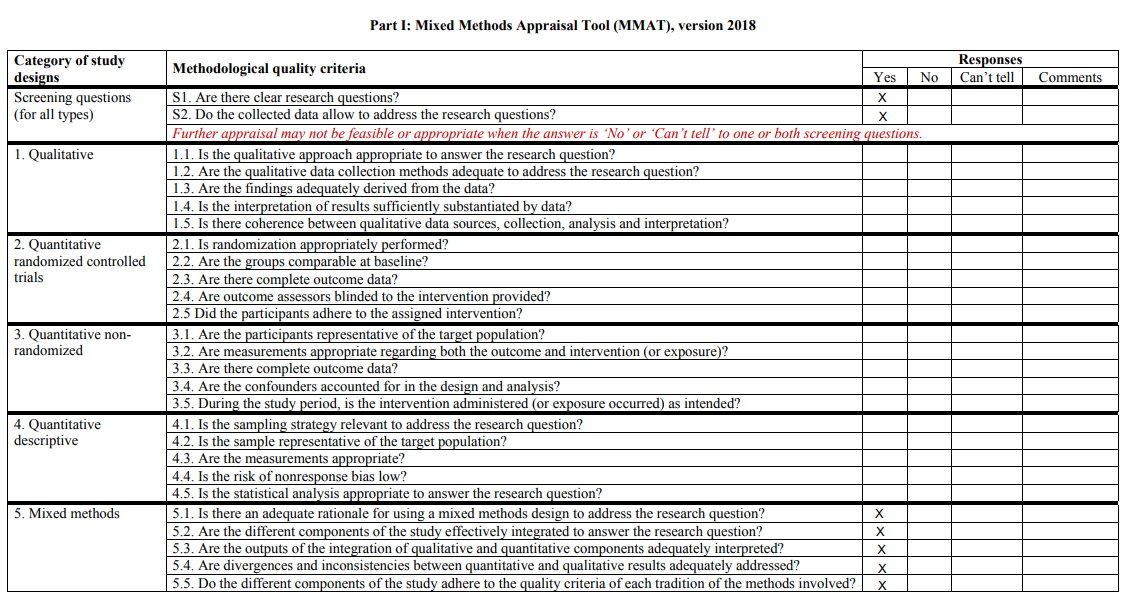


Creative dance: Potentiality for enhancing social functioning in frail seniors and young children (Rossberg Gempton *et al*. 1999)


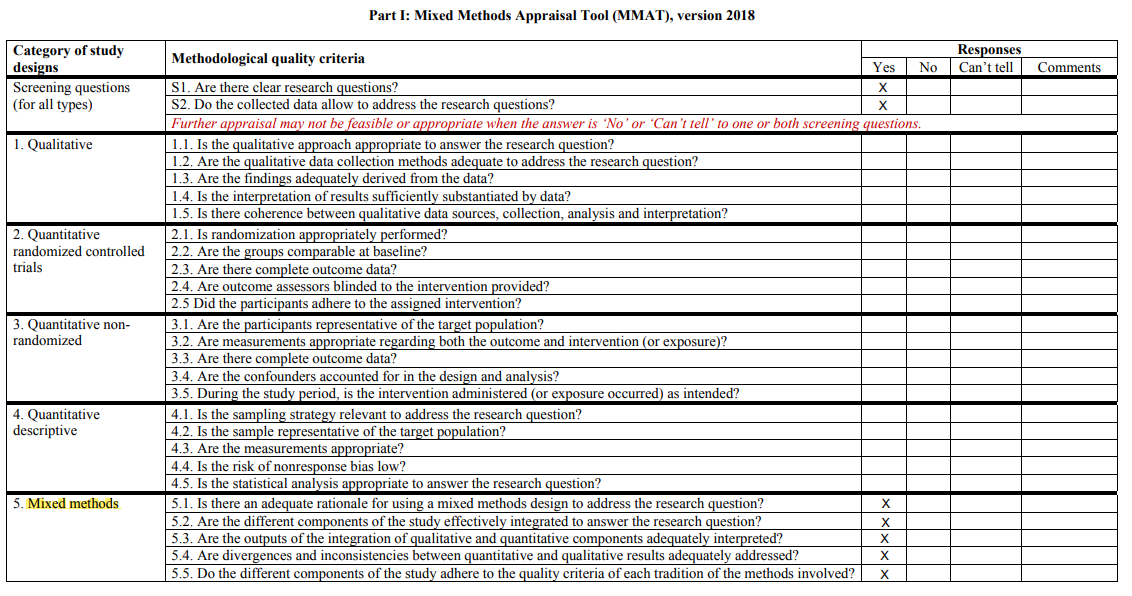


The Impact of an Intergenerational Dance Project on Older Adults’ Social and Emotional Well-Being (Douse *et al*. 2020)


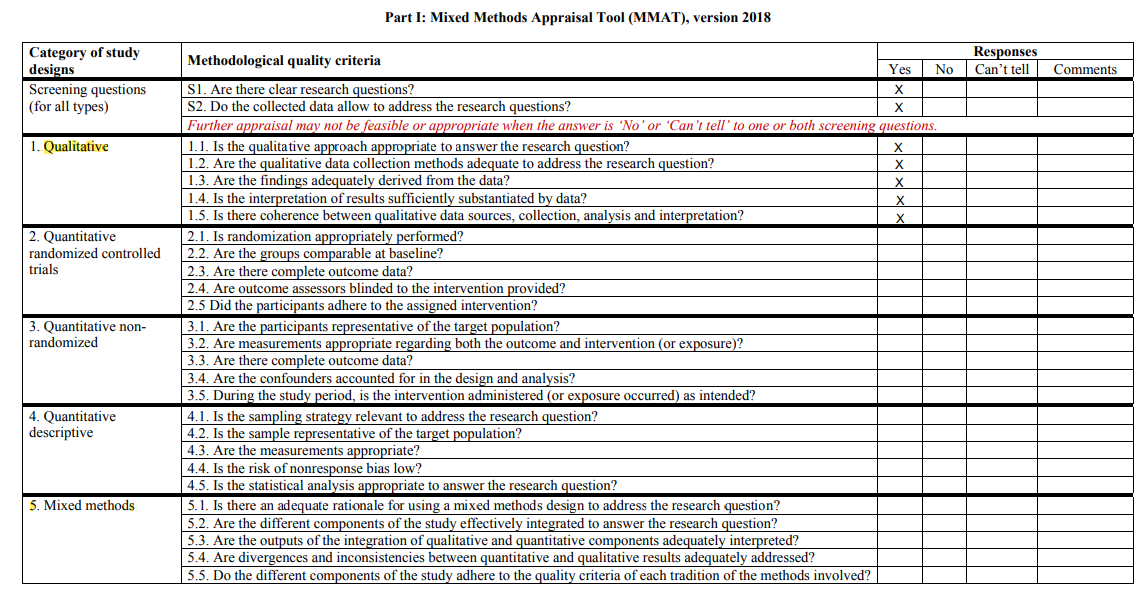


A case study of intergenerational relations through dance with profoundly deaf individuals (Sherman, 1997)


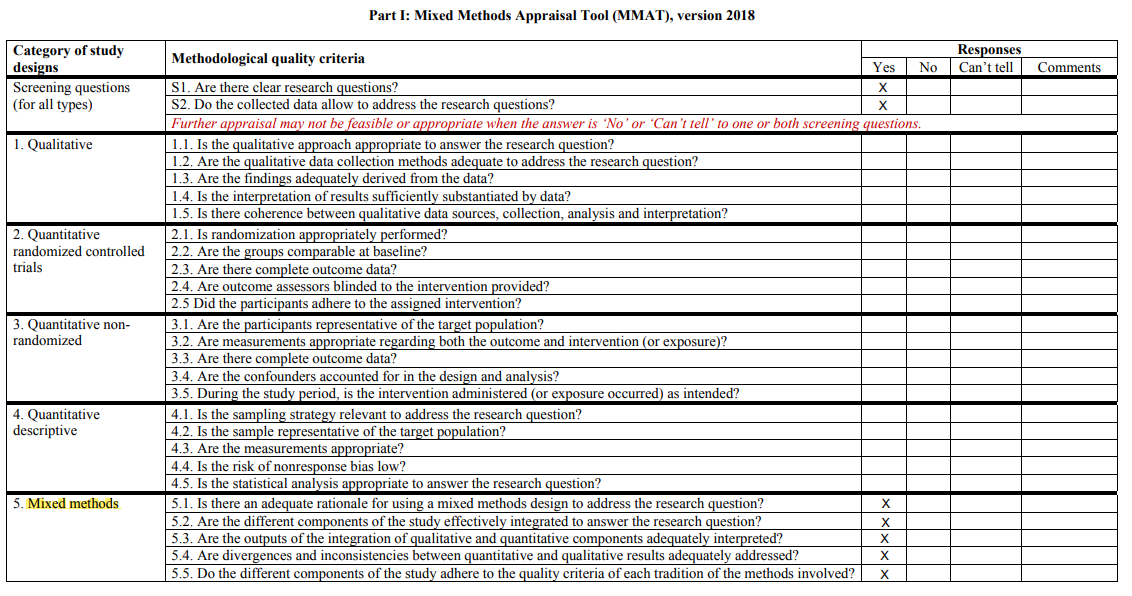


Playing remotely in times of crisis: A program to overcome social isolation (Brandao *et al*. 2022)


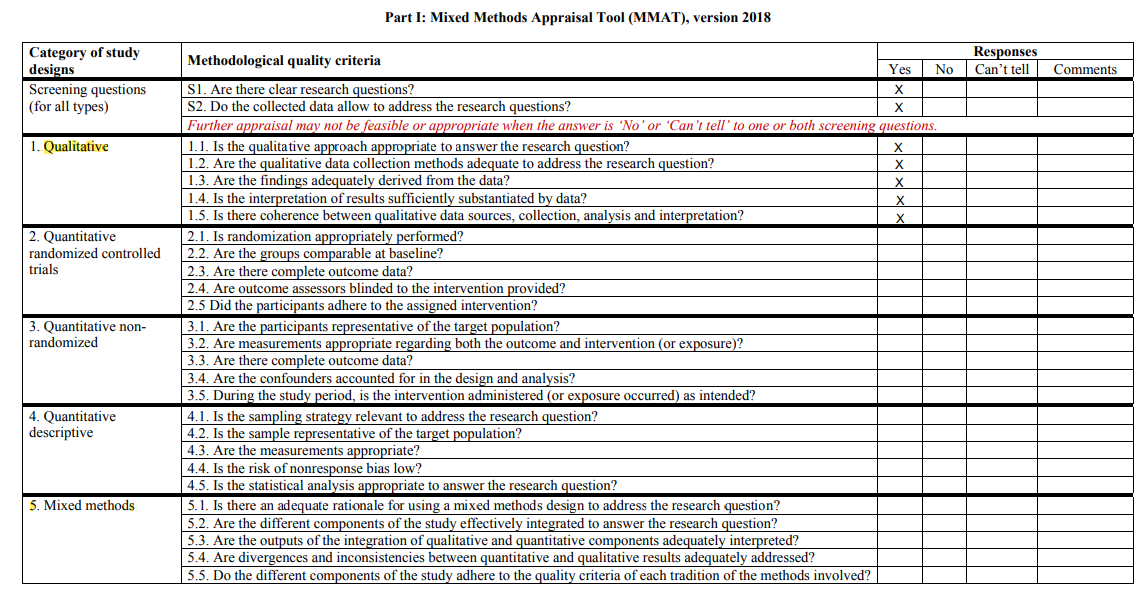


Qualitative inquiry of a community dance program for older adults in Singapore (Wu *et al*. 2023)


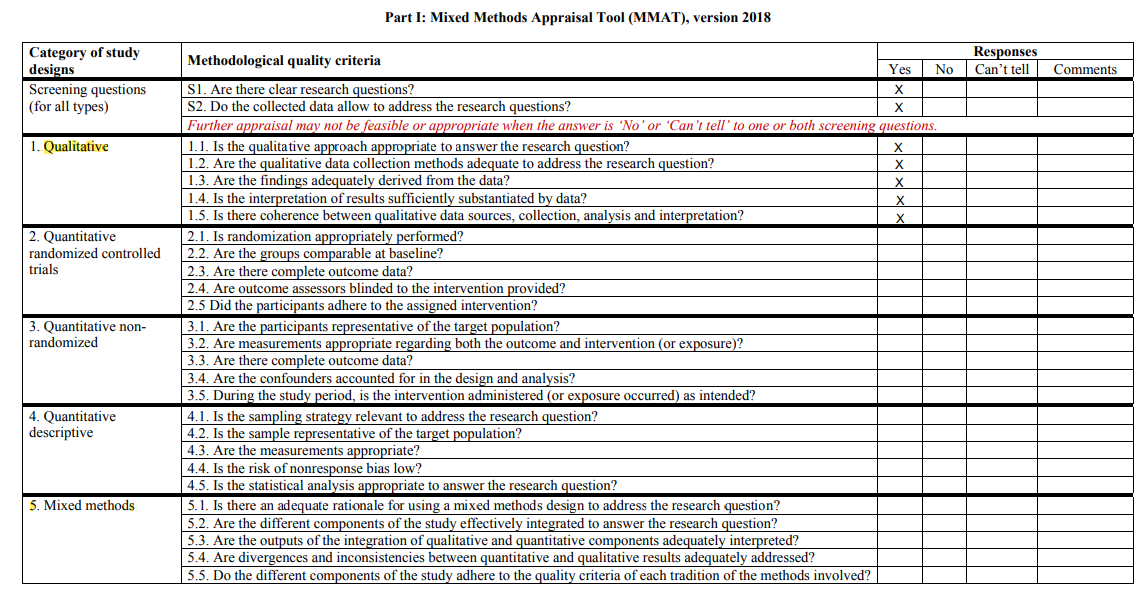


Sustainable Arts and Health: The Role of a University in Facilitating an Intergenerational, Interdisciplinary Community Arts Project (Farrer *et al*. 2022)


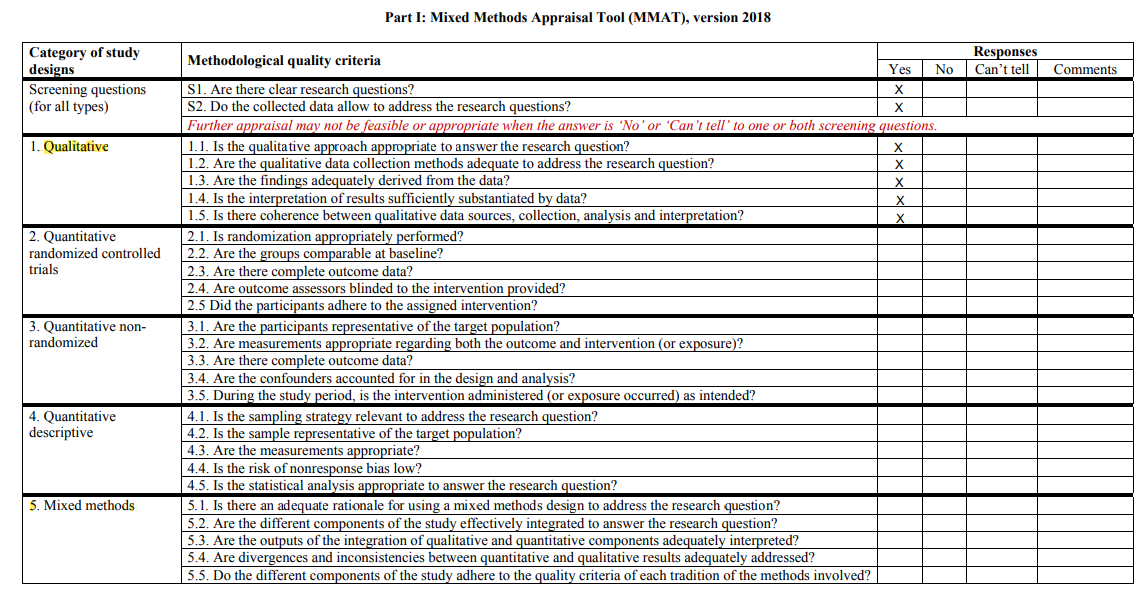


Understanding the impact of an intergenerational arts and health project: a study into the psychological well-being of participants, carers and artists (Jenkins *et al*. 2021)


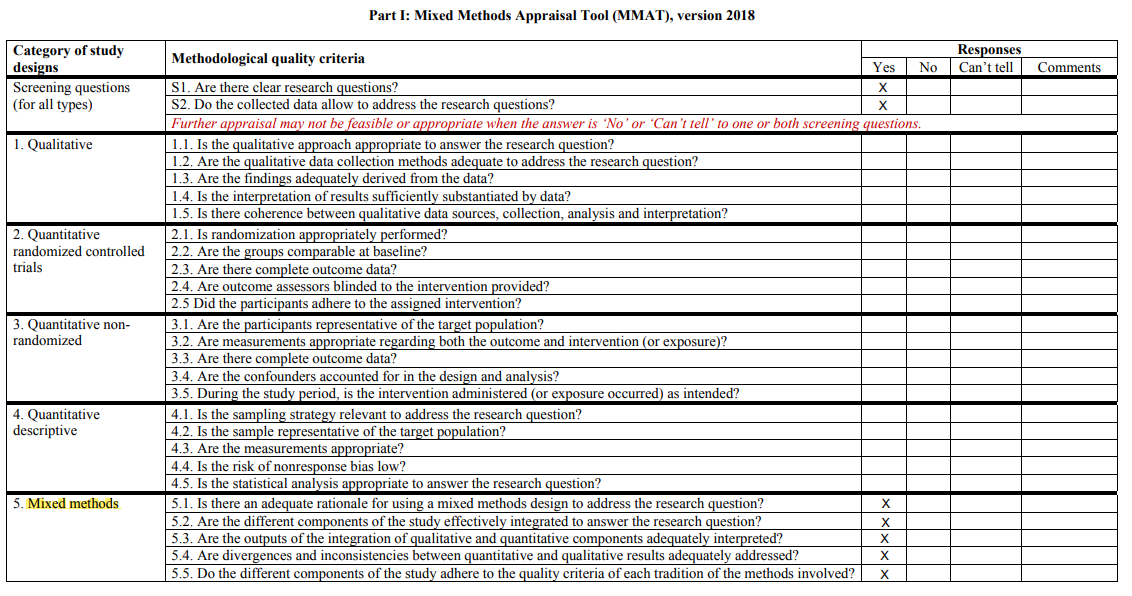


"Having Our Say" Exploring the Processes and Feasibility of a Community-Based Participatory Intergenerational Physical Activity Program for Grandparents Raising Grandchildren (Young, 2014)
