# Supplementary material for: A comprehensive scoping review of intergenerational dance programmes for cohorts with a generational gap
Source: PLoS One. 2024 Dec 19;19(12):e0311564. doi: 10.1371/journal.pone.0311564 (PMC11658520; doi:10.1371/journal.pone.0311564)
Supplement: Attachment — Submitted filename: Response to Reviewers.docx [file pone.0311564.s006.docx]

| **Reviewer 1 comment** | **Response** |
| --- | --- |
| Thank you to the authors for addressing all of the comments thoroughly and thoughtfully. Only one edit was not addressed completely: Table 2 the definition of IG. In row 3 Morita and Kobayashi, 2012, the description of the program states IG. After the abbreviation IG please add '*', then the definition below the table should read "IG = intergenerational". The rest of the explanation may/may not need to be included if you wish as this is described in the text. Great job on this lovely paper. | Thank you very much for your kind feedback.  Table 2 row three has been amended to include “**”, with "IG = intergenerational" included below. |
